# Association between lipid accumulation product and chronic obstructive pulmonary disease: a cross-sectional study based on U.S. adults

**DOI:** 10.3389/fnut.2024.1517108

**Published:** 2025-01-10

**Authors:** Xingshi Hua, Ying Liu, Xiaoyu Xiao

**Affiliations:** ^1^Liaoning University of Traditional Chinese Medicine, Shenyang, China; ^2^Liaoning University of Traditional Chinese Medicine Affiliated Second Hospital, Shenyang, China; ^3^Department of Pathology, The First Affiliated Hospital of Liaoning University of Traditional Chinese Medicine, Shenyang, China

**Keywords:** NHANES, COPD, LAP, obesity, cross-sectional study

## Abstract

**Background:**

Lipid Accumulation Product (LAP), which is derived from measurements of waist circumference and triglyceride (TG) levels, serves as a comprehensive indicator of lipid accumulation. Emerging research indicates that lipid accumulation dysfunction might significantly contribute to the pathogenesis of Chronic Obstructive Pulmonary Disease (COPD). Nevertheless, the investigation into the association between LAP and COPD risk is still insufficient, particularly in population-based research. This research intends to examine the possible correlation between LAP and the likelihood of developing COPD.

**Methods:**

This study, designed as a cross-sectional analysis, made use of data gathered from the National Health and Nutrition Examination Survey (NHANES) spanning the years 2017 to 2020, encompassing a total of 7,113 eligible participants. LAP, the exposure variable, was calculated using waist circumference and triglyceride concentration. COPD diagnosis was determined using participants’ self-reported information. To explore the association between LAP and COPD, multivariate logistic regression models were applied, and smoothing curve fitting was employed to examine any potential nonlinear patterns. Further analysis included stratified subgroup evaluations to assess how variables such as sex, smoking habits, and alcohol intake might impact the relationship between LAP and COPD.

**Results:**

The findings indicated a significant increase in COPD risk with each one-unit rise in ln LAP, as evidenced by an Odds Ratio (OR) of 1.16 [95% Confidence Interval (CI): 1.04–1.30, *p* < 0.01]. Furthermore, a quartile-based analysis revealed that individuals in the highest ln LAP category had a considerably higher likelihood of developing COPD compared to those in the lowest category, with an OR of 1.35 (95% CI: 1.04–1.75, *P* for trend <0.01). Furthermore, the smoothing curve fitting identified a nonlinear and positive association between ln LAP and COPD, suggesting a steeper increase in risk as ln LAP values rise. Subgroup analysis suggested that this association remained fairly consistent across various demographic groups.

**Conclusion:**

This study found a significant link between higher LAP levels and an elevated risk of COPD, with the association displaying a nonlinear pattern. As a marker of lipid accumulation abnormalities, LAP may serve as a valuable tool for assessing COPD risk and could inform strategies for early identification and targeted clinical management.

## 1 Introduction

As a condition characterized by a complex interplay of respiratory abnormalities, Chronic Obstructive Pulmonary Disease (COPD) primarily manifests as persistent airflow obstruction and heightened inflammation within the lung tissue (1, 2). It predominantly affects middle-aged and elderly populations (3, 4). In recent decades, COPD prevalence has been on an upward trend, largely driven by factors such as escalating environmental pollution and an aging global population, thereby creating a substantial economic strain on society (5, 6). The World Health Organization (WHO) forecasts that COPD prevalence will keep rising over the coming four decades, with annual deaths potentially exceeding 5.4 million by 2060 due to COPD and its associated complications (7, 8). Despite being a preventable and treatable major public health concern, there remain numerous shortcomings in the clinical management of COPD, including inadequate patient education and follow-up (9–12). These factors significantly impact the stability of the disease, leading to frequent exacerbations and disease progression. Additionally, symptom exacerbations not only affect patients’ daily activities and sleep quality but can also contribute to mental health issues (13, 14). The high prevalence, recurrent exacerbations, and protracted course of COPD place a heavy economic burden on patients, their families, and society (15, 16). Therefore, accelerating the standardization of COPD diagnosis, treatment, and prevention, while improving the precision and efficacy of clinical management, is of critical importance (17).

In the last 40 years, obesity rates have been on a consistent rise worldwide (18, 19). Numerous epidemiological studies have shown that obesity significantly enhances susceptibility to pulmonary diseases, suggesting that lipid accumulation or abnormal distribution resulting from obesity is a critical factor contributing to the development of respiratory conditions, including COPD (20, 21). Traditionally, obesity assessment relies on indicators such as Body Mass Index (BMI) to gauge overall body fat and Waist Circumference (WC) to measure abdominal fat (22–24). However, both methods present specific limitations (25). While BMI serves as a general indicator of obesity, it does not effectively differentiate between various body components, such as lean mass, water content, or visceral fat, which limits its accuracy in assessing the risk linked to specific health conditions (26). While WC can indicate abdominal obesity, it cannot accurately distinguish between visceral and subcutaneous fat, with visceral fat being closely linked to metabolic disorders (27). To better reflect the health impact of excessive fat accumulation, the Lipid Accumulation Product (LAP) was introduced. LAP combines WC and fasting triglyceride (TG) concentration, offering a more accurate assessment of changes in lipid accumulation (28).

Lipid Accumulation Product (LAP) is a cost-effective anthropometric measure that is easy to calculate and obtain, providing a novel perspective for understanding the impact of obesity on respiratory health (29, 30). Studies have demonstrated that LAP outperforms traditional indicators in predicting various health conditions, including heart-related diseases, type 2 diabetes, and metabolic abnormalities (31, 32). As a marker of lipid accumulation abnormalities, LAP can more effectively quantify the accumulation of visceral fat and its associated health risks (33). Therefore, exploring the association between LAP and COPD risk can provide a more comprehensive understanding of how lipid accumulation abnormalities may contribute to COPD progression.

Given this context, this study seeks to investigate how variations in lipid accumulation might relate to the occurrence of COPD, delving deeper into their potential connection. Using data from the National Health and Nutrition Examination Survey (NHANES), a nationally representative dataset, allows us to comprehensively explore this association within a diverse U.S. population. Our research focuses on examining how LAP is related to COPD risk, highlighting the interactions among lipid accumulation, obesity, and COPD. Our hypothesis posits that elevated LAP could increase the risk of developing COPD.

## 2 Methods

### 2.1 Study design and sample

In this cross-sectional analysis, we explored the relationship between LAP and COPD using data from the National Health and Nutrition Examination Survey (NHANES) spanning 2017 to 2020. NHANES employs a sophisticated sampling method, incorporating a multistage, stratified design to accurately represent the health status of the non-institutionalized U.S. population. Before participating in the survey, all individuals provided written informed consent, and the study was conducted under ethical guidelines approved by the relevant review board. Comprehensive details about the NHANES methodology, sampling strategies, and data collection procedures are available on the CDC’s NHANES website (https://www.cdc.gov/nchs/nhanes/).

**Figure 1:**
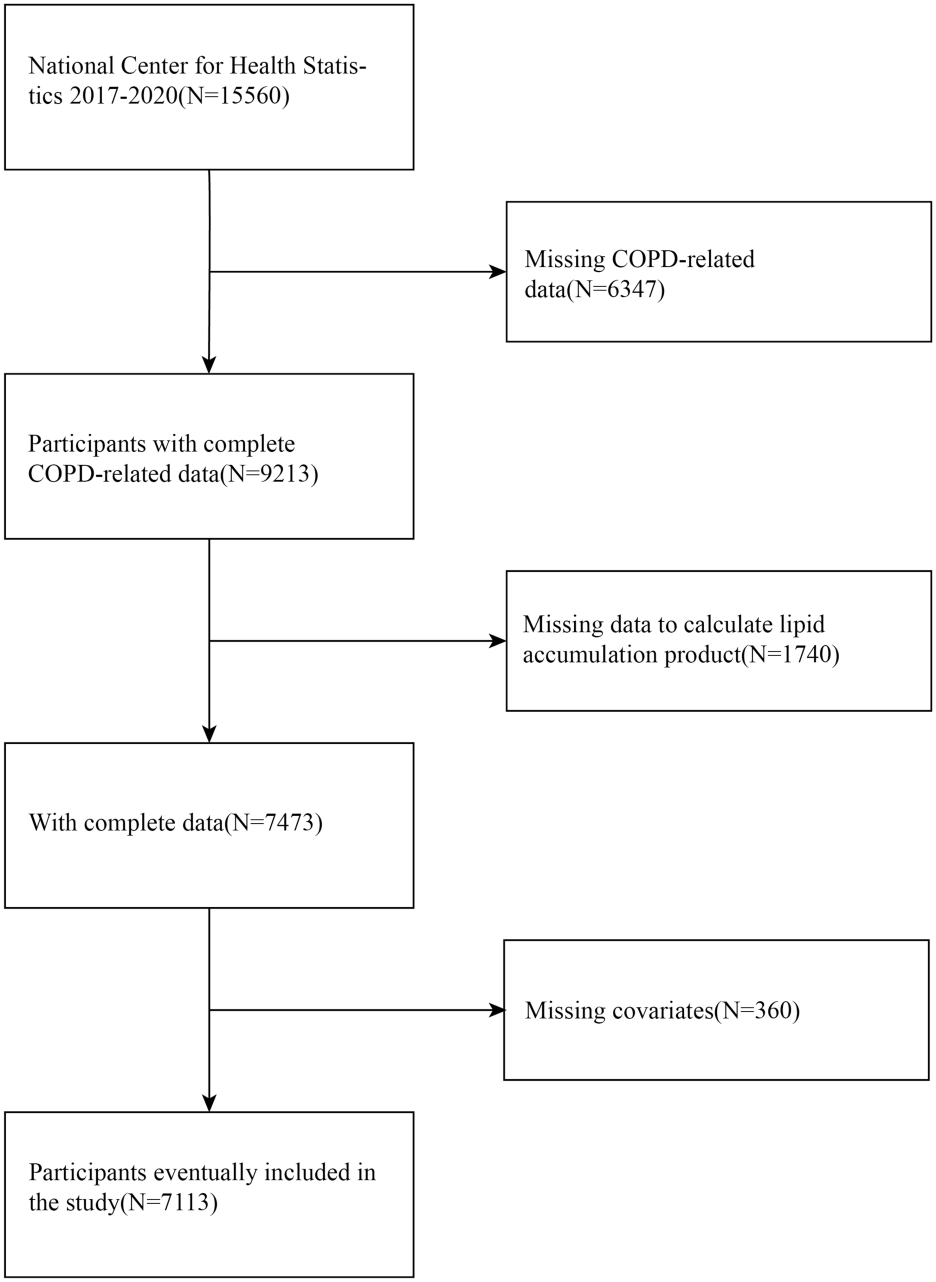
Flow chart of participant selection.

The initial analysis of this study encompassed 7,113 eligible participants (Figure 1). Participants were excluded if they met any of the following conditions: (1) those without information regarding a COPD diagnosis; (2) those missing necessary data for LAP calculation; and (3) those lacking data on key covariates, including smoking status and alcohol consumption.

### 2.2 Exposure variable: LAP

In this study, LAP was used as the primary exposure variable, with its calculation differing by sex. The calculation of LAP was performed using specific formulas tailored separately for males and females. In NHANES, anthropometric measurements were performed by health technicians who had received specialized training, using mobile examination centers for consistency. WC was specifically measured at the uppermost part of the iliac crest with the use of a measuring tape, and the values were documented in centimeters (cm).


$$\mathrm{LAP}=\left(WC\;\left(\mathrm{cm}\right)-58\right)\times TG\;\left(\mathrm{mmol}/L\right)\;\mathrm{for females}$$

$$\mathrm{LAP}=\left(WC\;\left(\mathrm{cm}\right)-65\right)\times TG\;\left(\mathrm{mmol}/L\right)\;\mathrm{for males}$$

### 2.3 Outcome variable: COPD

In this study, the diagnosis of COPD was obtained through self-reported questionnaires completed by participants. Participants were asked whether they had ever received a diagnosis of COPD, emphysema, or chronic bronchitis from a doctor or healthcare professional. This questionnaire-based method efficiently identifies COPD patients and has been widely used in related studies, demonstrating a certain level of reliability (34).

### 2.4 Covariates

This study took into account several covariates that could potentially affect the association between LAP and COPD, including gender, ethnicity, educational attainment, marital status, alcohol consumption, and smoking behavior. Marital status was categorized as married, cohabiting, and unmarried/single. Smoking status was classified into never smokers and smokers. Participants’ alcohol consumption was categorized into drinkers and non-drinkers, based on their self-reported alcohol intake. These covariates were adjusted for during data analysis to control for their potential impact on the association between LAP and COPD. Detailed definitions and measurement methods for each covariate can be found in the official NHANES documentation.

### 2.5 Statistical analyses

In this study, we performed statistical analyses using R software (v4.2.0) along with EmpowerStats 2.0 for thorough data evaluation. These analyses took full consideration of the NHANES complex sampling framework and applied sample weights to guarantee representativeness at the national level and enhance statistical reliability. For this analysis, statistical significance was determined using a threshold *p*-value of less than 0.05, indicating a high likelihood that the observed results were not due to random chance. To ensure the robustness of the findings, all procedures adhered to CDC guidelines and incorporated the NHANES sampling weights throughout the analysis.

Descriptive statistics were used to outline the baseline characteristics of the participants. Categorical variables were expressed as weighted proportions, while continuous variables were presented as weighted means with standard deviations (SD). Group differences were assessed using appropriate statistical methods for categorical and continuous data. Given the non-normal distribution of LAP, a natural logarithmic transformation (ln LAP) was applied to approximate normality for further analyses.

In order to thoroughly examine the connection between ln LAP and COPD, we utilized weighted multivariate logistic regression models. This method facilitated a detailed exploration of the potential association while controlling for potential confounders to strengthen the validity of our analysis. Model I did not adjust for any covariates, providing the crude association between ln LAP and COPD. Model II included adjustments for demographic variables such as sex, race, and age. To further account for potential confounding factors, Model III incorporated additional adjustments for educational attainment, marital status, alcohol intake, and smoking behavior. To assess the association between LAP and COPD, odds ratios (ORs) were estimated for each model. Subsequently, the precision of these estimates was gauged by calculating the 95% confidence intervals (CIs). Furthermore, a smoothing curve fitting method was utilized to explore potential nonlinear relationships between ln LAP and the risk of COPD, providing a deeper insight into their interaction. Subgroup analyses and interaction tests were conducted across various populations, particularly focusing on stratification by sex, smoking status, and alcohol consumption, to pinpoint potentially vulnerable subgroups.

## 3 Results

### 3.1 Baseline characteristics of participants

The distribution of demographic factors and additional covariates among the participants, categorized by their COPD status, is detailed in Table 1. Within the cohort, 7,113 individuals were examined, and 632 of them were confirmed to have COPD. Among the COPD patients, 48.10% were male. After adjusting for weights, the average age of participants diagnosed with COPD was 60.11 ± 15.18 years. This age is significantly higher compared to the mean age of 49.69 ± 17.22 years recorded for individuals without COPD (*p* < 0.001). Furthermore, the COPD group exhibited a notably higher proportion of smokers, alcohol consumers, and individuals who were single (*p* < 0.001). Statistical analysis also revealed differences in other covariates, including race and education level, when comparing the two groups. Importantly, the ln LAP values in individuals diagnosed with COPD were markedly elevated compared to those in the non-COPD group, further implying a possible link between lipid accumulation and the development of COPD.

**Table 1:** Baseline characteristics of participants, categorized by COPD status.

Characteristic	Non-COPD *N* = 6,481 (91.1%)	COPD *N* = 632 (8.9%)	*P*-value
Age (year)	49.69 ± 17.22	60.11 ± 15.18	<0.001
Triglycerides (mmol/L)	1.56 ± 1.23	1.60 ± 0.95	0.002
BMI	29.87 ± 7.13	31.54 ± 8.68	<0.001
WC	100.72 ± 16.90	106.90 ± 18.38	<0.001
Total cholesterol (mmol/L)	4.82 ± 1.06	4.65 ± 1.08	<0.001
LAP	64.57 ± 62.04	76.32 ± 57.81	<0.001
Ln LAP	3.84 ± 0.84	4.06 ± 0.79	<0.001
**Gender (%)**			0.571
Male	3,194 (49.28%)	304 (48.10%)	
Female	3,287 (50.72%)	328 (51.90%)	
**Race (%)**			<0.001
Mexican American	827 (12.76%)	27 (4.27%)	
Other Hispanic	684 (10.55%)	45 (7.12%)	
Non-Hispanic White	2,234 (34.47%)	360 (56.96%)	
Non-Hispanic Black	1,664 (25.68%)	132 (20.89%)	
Non-Hispanic Asian	785 (12.11%)	17 (2.69%)	
Other Race - Including Multi-Racial	287 (4.43%)	51 (8.07%)	
**Alcohol (%)**			0.001
Drinks	5,895 (90.96%)	599 (94.78%)	
Never	586 (9.04%)	33 (5.22%)	
**Hypertension (%)**			<0.001
Yes	2,351 (36.28%)	362 (57.28%)	
No	4,130 (63.72%)	270 (42.72%)	
**Diabetes (%)**			<0.001
Yes	1,076 (16.60%)	203 (32.12%)	
No	5,405 (83.40%)	429 (67.88%)	
**Smoking status (%)**			<0.001
Yes	2,543 (39.24%)	463 (73.26%)	
No	3,938 (60.76%)	169 (26.74%)	
Marital status (%)			<0.001
Married/Living with partners	3,852 (59.44%)	315 (49.84%)	
Widowed/Divorced/Separated	1,346 (20.77%)	224 (35.44%)	
Never married	1,283 (19.80%)	93 (14.72%)	
**Education level (%)**			<0.001
Less than 9th grade	443 (6.84%)	39 (6.17%)	
9-11th grade	663 (10.23%)	94 (14.87%)	
High school graduate	1,531 (23.62%)	198 (31.33%)	
Some college or AA degree	2,134 (32.93%)	231 (36.55%)	
College graduate or above	1710 (26.38%)	70 (11.08%)	

### 3.2 Association between LAP and COPD risk

The analysis, accounting for all relevant covariates, indicated that with every one-unit increase in ln LAP, the risk of developing COPD increased by 16%. This association was statistically significant, as demonstrated by an odds ratio (OR) of 1.16, with a 95% confidence interval (CI) ranging from 1.04 to 1.30 (*p* < 0.01). This result suggests a strong association between elevated LAP levels and a higher risk of developing COPD, highlighting the potential impact of lipid accumulation on the disease’s onset. Further analysis, categorizing ln LAP into quartiles, indicated that participants within Q4 had a significantly greater risk of COPD when compared to those in Q1. This elevated risk corresponded to an OR of 1.35, with a 95% CI of 1.04 to 1.75, along with a statistically significant trend (*P* for trend <0.01) as detailed in Table 2.

**Table 2:** Multivariable logistic regression models for the association between LAP and COPD.

OR (95%CI), *P*-value			
	Model I	Model II	Model III
COPD			
Ln LAP Continuous	1.38 (1.25, 1.53) <0.0001	1.22 (1.09, 1.37) 0.0005	1.16 (1.04, 1.30) 0.0089
Categories			
Quartile1	Ref	Ref	Ref
Quartile2	1.40 (1.09, 1.82) 0.0096	1.05 (0.80, 1.37) 0.7268	1.12 (0.85, 1.47) 0.4252
Quartile3	1.65 (1.28, 2.12) <0.0001	1.19 (0.91, 1.54) 0.1996	1.17 (0.89, 1.53) 0.2571
Quartile4	2.02 (1.58, 2.57) <0.0001	1.46 (1.13, 1.88) 0.0035	1.35 (1.04, 1.75) 0.0234
*P* for trend	1.44 (1.27, 1.62) <0.0001	1.24 (1.09, 1.41) 0.0013	1.17 (1.02, 1.33) 0.0200

### 3.3 Analysis of curve fitting

**Figure 2:**
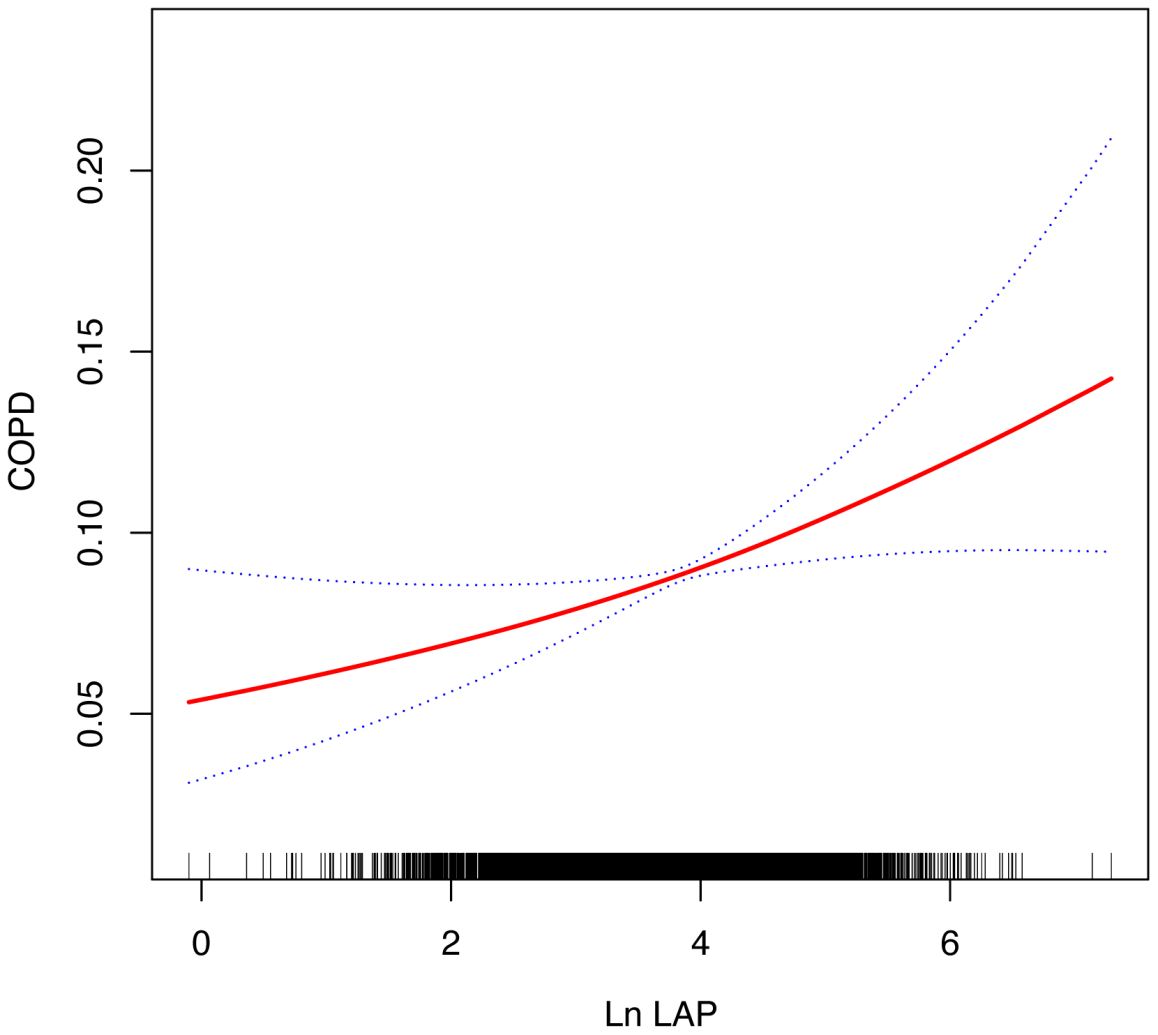
The smoothed curve between LAP and COPD is depicted in red, with blue bars representing the 95% CI.

The smoothing curve fitting analysis offered deeper insights, revealing that the relationship between ln LAP and COPD risk does not follow a simple linear pattern, suggesting a more complex interaction. As shown in Figure 2, the risk of COPD exhibited an accelerating upward trend with increasing ln LAP, particularly at higher ln LAP levels where the risk significantly increased. This nonlinear relationship indicates that the effect of ln LAP on COPD risk is not a simple linear increase; rather, the risk of COPD becomes more pronounced at elevated LAP levels. The area shaded in blue illustrates the 95% confidence interval (95% CI), further confirming that this nonlinear trend is statistically significant.

### 3.4 Subgroup analysis

A set of subgroup analyses and interaction tests was carried out, incorporating different covariates, to assess how robust the association between LAP and COPD is. Additionally, this approach helped identify any significant variations within specific subgroups (Table 3). The analysis uncovered that, in the majority of subgroups examined, higher LAP levels were significantly linked with an increased prevalence of COPD. Although the association between LAP and COPD risk was more pronounced in certain specific populations, the interaction *p* values for most subgroups did not show significant differences. This suggests that the relationship between LAP and COPD risk is relatively stable across different populations, with no apparent interactions identified. This consistency indicates that LAP, as a potential risk factor, demonstrates a uniform association with COPD risk across various subgroups.

**Table 3:** Subgroup Stratified Analysis of LAP and COPD with Adjustments from Model III.

Subgroup analysis	COPD, OR(95%CI), *P*-value	*P* for interaction
**Gender (%)**		0.8670
Male	1.16 (1.00, 1.35) 0.0559	
Female	1.18 (1.00, 1.40) 0.0466	
**Race (%)**		0.4916
Mexican American	0.70 (0.40, 1.23) 0.2143	
Other Hispanic	1.21 (0.80, 1.83) 0.3638	
Non-Hispanic White	1.15 (0.98, 1.34) 0.0806	
Non-Hispanic Black	1.30 (1.03, 1.66) 0.0298	
Non-Hispanic Asian	0.96 (0.50, 1.85) 0.9120	
Other Race - Including Multi-Racial	1.11 (0.71, 1.73) 0.6591	
**Education level (%)**		0.8236
Less than 9th grade	1.04 (0.60, 1.82) 0.8824	
9-11th grade	1.09 (0.79, 1.50) 0.5956	
High school graduate	1.08 (0.88, 1.33) 0.4606	
Some college or AA degree	1.25 (1.04, 1.49) 0.0166	
College graduate or above	1.26 (0.89, 1.77) 0.1966	
**Marital status (%)**		0.8096
Married/Living with partners	1.13 (0.96, 1.32) 0.1464	
Widowed/Divorced/Separated	1.22 (0.99, 1.50) 0.0602	
Never married	1.12 (0.86, 1.45) 0.4017	
**Alcohol (%)**		0.8717
Drinks	1.17 (1.04, 1.31) 0.0086	
Never	1.11 (0.60, 2.05) 0.7423	
**Smoking status (%)**		0.1091
Yes	1.10 (0.97, 1.26) 0.1448	
No	1.35 (1.09, 1.69) 0.0067	

## 4 Discussion

In this study, we investigated the link between the LAP index and COPD prevalence, utilizing data sourced from the NHANES database. The findings indicated a significant positive link between higher LAP levels and a greater risk of COPD, reinforcing the potential involvement of lipid accumulation abnormalities in the development of COPD.

Firstly, the model analysis showed that with every one-unit increment in ln LAP, the odds of developing COPD rose by approximately 16%, as indicated by an OR of 1.16 (95% CI: 1.04–1.30, *p* < 0.01). This result points to a distinct positive association between LAP levels and COPD risk. Additionally, the quartile-based assessment supported this observation, indicating that participants in the highest ln LAP quartile had a substantially elevated likelihood of COPD relative to those in the lowest quartile. These outcomes imply that lipid accumulation is closely linked to COPD risk, suggesting that monitoring and managing LAP could be crucial in the prevention and treatment strategies for COPD.

Additionally, the study found that the relationship between LAP and COPD may exhibit a non-linear positive correlation. The analysis using smooth curve fitting indicated that the relationship between ln LAP levels and COPD risk is non-linear, showing a marked increase in COPD risk, especially when ln LAP levels reach the higher range. This further emphasizes the importance of controlling lipid accumulation in reducing COPD risk. Moreover, findings derived from subgroup analyses and interaction tests indicated that this association remains fairly consistent across various populations, showing no significant impact from factors such as gender, age, race, or other typical covariates. This finding suggests that lipid accumulation abnormalities may exhibit a consistent association with COPD incidence across diverse groups.

To our knowledge, this is the first study based on NHANES data from 2017 to 2020 that assesses the association between LAP index and COPD risk. Previous studies primarily relied on earlier NHANES data to explore the relationships between other common indicators or specific nutrients and COPD (35).

Research indicates that obesity significantly impacts lung function, with obesity-related indicators showing a negative correlation with pulmonary function (36–38). This effect stems from how adipose tissue impacts the mechanical behavior of the respiratory system; for instance, abdominal obesity can reduce lung compliance and substantially decrease lung volume, while visceral fat may alter diaphragmatic structure and restrict its movement, thereby impairing lung function (39, 40).

As a comprehensive lipid accumulation indicator, LAP incorporates waist circumference and fasting triglyceride (TG), both closely related to lipid accumulation. While WC and TG may individually contribute to COPD risk, LAP could offer a more comprehensive reflection of both abdominal obesity and dyslipidemia. Elevated LAP typically reflects visceral fat accumulation and metabolic dysfunction, with increased TG levels often associated with inflammatory responses and the onset of various chronic diseases (41, 42). In the pathogenesis of COPD, lipid accumulation abnormalities and systemic inflammation are recognized as significant triggering factors (43, 44). Additionally, the higher prevalence of comorbid diabetes within the COPD group suggests that dyslipidemia may interact with metabolic disorders to exacerbate COPD severity (45, 46). Thus, LAP serves as a crucial indicator of lipid accumulation in relation to COPD. Earlier clinical investigations have examined how LAP relates to different disease outcomes, yet research specifically addressing COPD remains limited. Studies have shown that COPD patients often exhibit lipid accumulation abnormalities (47, 48). A study involving a Spanish population found that among 1,500 subjects, 48.3% of COPD patients had dyslipidemia (49). This indicates that disruptions in lipid accumulation could be a key factor in the pathogenesis of COPD.

Chronic inflammation and smoking are recognized as major mechanisms leading to lipid accumulation abnormalities in patients with COPD. TG and other lipids are transported from the liver to the bloodstream via lipoproteins. Once these lipid-rich particles are taken up by macrophages, they can lead to lipid deposition, subsequently triggering inflammatory responses (50). Chronic inflammation and lipid accumulation abnormalities play a dual role in COPD. Pro-inflammatory cytokines can elevate circulating TG levels, while inflammation suppression may lower these levels (51, 52). This phenomenon further underscores the complex interplay between lipid accumulation and inflammation, highlighting their central roles in the pathogenesis of COPD. These mechanisms may partly explain the observed association between elevated LAP and increased COPD risk in this study, as LAP reflects both TG levels and abdominal obesity, which are closely linked to systemic inflammation.

Smoking is recognized as a major risk factor for COPD and has a profound effect on lipid accumulation (53). Research has demonstrated that smoking can increase the levels of circulating lipids in the bloodstream, contributing to an altered lipid profile that may include higher concentrations of harmful fats (54). Earlier research has suggested that exposure to cigarette smoke can trigger the production of fatty acids within epithelial cells of the respiratory tract. This process subsequently impacts autophagy, the release of inflammatory mediators, and cell apoptosis (55, 56). Chronic exposure to cigarette smoke disrupts fatty acid accumulation homeostasis, a factor considered crucial in the onset and progression of COPD (57, 58). Furthermore, the increased fatty acid oxidation (FAO) caused by cigarette smoke exposure may exacerbate cellular damage through mitochondrial injury and the accumulation of reactive oxygen species (ROS), implying that such metabolic changes are crucial for preserving lipid accumulation homeostasis in the body (59, 60).

Daily symptoms experienced by patients with COPD, such as cough and chest tightness, can further impact their exercise tolerance, potentially increasing the risk of lipid accumulation abnormalities (61). Additionally, the use of glucocorticoids, particularly during acute exacerbations, may lead to adverse outcomes such as obesity and lipid accumulation abnormalities due to elevated glucocorticoid levels in the body (62). Evidence from a large-scale population study has revealed that brief, low-dose glucocorticoid treatment can have a notable impact on lipid accumulation levels (63). The prevalence of elevated LAP values among COPD patients is gradually increasing, and these abnormalities may be closely associated with poor prognosis and systemic complications (64). Recent studies have increasingly emphasized that COPD is not solely a pulmonary disease; it often accompanies extrapulmonary manifestations such as cardiovascular disease, malnutrition, and metabolic disorders (65). Dysregulation of lipid accumulation plays a significant role in these processes (49). Furthermore, lipid accumulation abnormalities not only exacerbate the risk of COPD but may also trigger systemic inflammation through increased oxidative stress and chronic inflammatory responses, further worsening the condition (66). Therefore, LAP has the potential to be an effective supplementary indicator for evaluating disease severity in individuals with COPD and may guide early identification of high-risk patients and tailored interventions to mitigate lipid accumulation abnormalities. However, to fully understand the underlying mechanisms, additional research involving larger cohorts and prospective study designs is required.

This study possesses certain strengths. Firstly, it makes use of nationally representative sampling data, allowing the study to effectively examine how LAP relates to COPD risk among diverse population groups. Secondly, the large sample size not only improves the precision of the statistical analysis but also supplies sufficient data to investigate how LAP correlates with COPD across different subgroups, revealing variations in this association according to demographic factors, including gender, age groups, and smoking habits. Nevertheless, this research has its constraints. Primarily, due to the study’s cross-sectional nature, identifying a clear causal link between the variables remains challenging. This means that while an association between LAP and COPD has been observed, we cannot determine whether changes in LAP directly lead to an increased risk of COPD. Second, due to data limitations, we were unable to include all potential covariates, which may result in unmeasured confounding bias. Additionally, the reliance on self-reported COPD diagnosis may introduce recall bias and potential misclassification, further suggesting that our findings should be interpreted with caution.

Therefore, future research should consider adopting longitudinal designs and more comprehensive data collection methods, which would allow for a deeper understanding of how lipid accumulation may influence the development and progression of COPD.

## 5 Conclusion

This study’s findings highlight a significant relationship between lipid accumulation levels and the risk of COPD. Notably, elevated LAP is positively correlated with the prevalence of COPD, exhibiting a dose–response relationship. Furthermore, the smooth curve fitting analysis reveals a potential nonlinear positive correlation between LAP and COPD. Overall, this work provides insights into the interplay between LAP and COPD, highlighting the importance of managing lipid accumulation levels as a strategy for the prevention and management of COPD. These findings establish a scientific foundation for formulating public health strategies and set the stage for future research to explore the role of lipid accumulation in COPD pathogenesis.

## Data Availability

Publicly available datasets were analyzed in this study. This data can be found at: data repository: [National Health and Nutrition Examination Survey (NHANES)] direct link to data: https://www.cdc.gov/nchs/nhanes/index.htm.

## References

[1] Lahousse L Tiemeier H Ikram MA Brusselle GG. Chronic obstructive pulmonary disease and cerebrovascular disease: a comprehensive review. Respir Med. (2015) 109:1371–80. doi: 10.1016/j.rmed.2015.07.014, PMID: 26342840

[2] Christenson SA Smith BM Bafadhel M Putcha N. Chronic obstructive pulmonary disease. Lancet. (2022) 399:2227–42. doi: 10.1016/S0140-6736(22)00470-6 35533707

[3] Safiri S Carson-Chahhoud K Noori M Nejadghaderi SA Sullman MJM Heris JA. Burden of chronic obstructive pulmonary disease and its attributable risk factors in 204 countries and territories, 1990-2019: results from the global burden of disease study 2019. BMJ. (2022) 378:e069679. doi: 10.1136/bmj-2021-069679, PMID: 35896191 PMC9326843

[4] Soriano JB Alfageme I Miravitlles M, Lucas P de, Soler-Cataluna JJ Garcia-Rio F Casanova C Gonzalez-Moro JMR. Prevalence and determinants of COPD in Spain: EPISCAN II. Arch Bronconeumol (2021) 57:61–69. doi: 10.1016/j.arbres.2020.07.024, PMID: 32950310

[5] World Health Organization. (2024). Chronic obstructive pulmonary disease (COPD). Available at: https://www.who.int/news-room/fact-sheets/detail/chronic-obstructive-pulmonary-disease-(copd) (Accessed September 24, 2024).

[6] Manisalidis I Stavropoulou E Stavropoulos A Bezirtzoglou E. Environmental and health impacts of air pollution: a review. Front Public Health. (2020) 8:14. doi: 10.3389/fpubh.2020.00014, PMID: 32154200 PMC7044178

[7] Lozano R Naghavi M Foreman K Lim S Shibuya K Aboyans V. Global and regional mortality from 235 causes of death for 20 age groups in 1990 and 2010: a systematic analysis for the global burden of disease study 2010. Lancet. (2012) 380:2095–128. doi: 10.1016/S0140-6736(12)61728-0, PMID: 23245604 PMC10790329

[8] World Health Organization. (2024). The top 10 causes of death. Available at: https://www.who.int/news-room/fact-sheets/detail/the-top-10-causes-of-death (Accessed September 24, 2024).

[9] Chetta A Olivieri D. The COPD assessment test in the evaluation of chronic obstructive pulmonary disease exacerbations. Expert Rev Respir Med. (2012) 6:373–5. doi: 10.1586/ers.12.37, PMID: 22971062

[10] Spruit MA Singh SJ Garvey C ZuWallack R Nici L Rochester C. An official American Thoracic Society/European Respiratory Society statement: key concepts and advances in pulmonary rehabilitation. Am J Respir Crit Care Med. (2013) 188:e13–64. doi: 10.1164/rccm.201309-1634ST, PMID: 24127811

[11] Cameron-Tucker HL Wood-Baker R Owen C Joseph L Walters EH. Chronic disease self-management and exercise in COPD as pulmonary rehabilitation: a randomized controlled trial. Int J Chron Obstruct Pulmon Dis. (2014) 9:513–23. doi: 10.2147/COPD.S58478, PMID: 24876771 PMC4037329

[12] Xiong T Bai X Wei X Wang L Li F Shi H. Exercise rehabilitation and chronic respiratory diseases: effects, mechanisms, and therapeutic benefits. Int J Chron Obstruct Pulmon Dis. (2023) 18:1251–66. doi: 10.2147/COPD.S408325, PMID: 37362621 PMC10289097

[13] Volpato E Toniolo S Pagnini F Banfi P. The relationship between anxiety, depression and treatment adherence in chronic obstructive pulmonary disease: a systematic review. Int J Chron Obstruct Pulmon Dis. (2021) 16:2001–21. doi: 10.2147/COPD.S313841, PMID: 34262270 PMC8275112

[14] Sampol J Miravitlles M Sáez M Pallero M Sampol G Ferrer J. Poor sleep quality, COPD severity and survival according to CASIS and Pittsburgh questionnaires. Sci Rep. (2023) 13:18656. doi: 10.1038/s41598-023-45717-9, PMID: 37907621 PMC10618283

[15] Zafari Z Li S Eakin MN Bellanger M Reed RM. Projecting long-term health and economic burden of COPD in the United States. Chest. (2021) 159:1400–10. doi: 10.1016/j.chest.2020.09.255, PMID: 33011203

[16] Akazawa M Halpern R Riedel AA Stanford RH Dalal A Blanchette CM. Economic burden prior to COPD diagnosis: a matched case-control study in the United States. Respir Med. (2008) 102:1744–52. doi: 10.1016/j.rmed.2008.07.009, PMID: 18760581

[17] Singh D Agusti A Anzueto A Barnes PJ Bourbeau J Celli BR. Global strategy for the diagnosis, management, and prevention of chronic obstructive lung disease: the GOLD science committee report 2019. Eur Respir J. (2019) 53:1900164. doi: 10.1183/13993003.00164-2019, PMID: 30846476

[18] NCD Risk Factor Collaboration (NCD-RisC). Worldwide trends in body-mass index, underweight, overweight, and obesity from. To 2016: a pooled analysis of 2416 population-based measurement studies in 128·9 million children, adolescents, and adults. Lancet. (1975) 390:2627–42. doi: 10.1016/S0140-6736(17)32129-3, PMID: 29029897 PMC5735219

[19] Ng M Fleming T Robinson M Thomson B Graetz N Margono C. Global, regional, and national prevalence of overweight and obesity in children and adults during 1980-2013: a systematic analysis for the global burden of disease study 2013. Lancet. (2014) 384:766–81. doi: 10.1016/S0140-6736(14)60460-8, PMID: 24880830 PMC4624264

[20] Svartengren M Cai G-H Malinovschi A Theorell-Haglöw J Janson C Elmståhl S. The impact of body mass index, central obesity and physical activity on lung function: results of the EpiHealth study. ERJ Open Res. (2020) 6:00214–2020. doi: 10.1183/23120541.00214-2020, PMID: 33263030 PMC7682662

[21] Dixon AE Peters U. The effect of obesity on lung function. Expert Rev Respir Med. (2018) 12:755–67. doi: 10.1080/17476348.2018.1506331, PMID: 30056777 PMC6311385

[22] Gastaldelli A Cusi K Fernandez Lando L Bray R Brouwers B Rodriguez A. Effect of tirzepatide versus insulin degludec on liver fat content and abdominal adipose tissue in people with type 2 diabetes (SURPASS-3 MRI): a substudy of the randomised, open-label, parallel-group, phase 3 SURPASS-3 trial. Lancet Diabetes Endocrinol. (2022) 10:393–406. doi: 10.1016/S2213-8587(22)00070-5, PMID: 35468325

[23] Kim NH Park Y Kim NH Kim SG. Weight-adjusted waist index reflects fat and muscle mass in the opposite direction in older adults. Age Ageing. (2021) 50:780–6. doi: 10.1093/ageing/afaa208, PMID: 33035293

[24] Yuan L Chang M Wang J. Abdominal obesity, body mass index and the risk of frailty in community-dwelling older adults: a systematic review and meta-analysis. Age Ageing. (2021) 50:1118–28. doi: 10.1093/ageing/afab039 33693472

[25] Smyth S Heron A. Diabetes and obesity: the twin epidemics. Nat Med. (2006) 12:75–80. doi: 10.1038/nm0106-75, PMID: 16397575

[26] Bray GA. Beyond BMI. Nutrients. (2023) 15:2254. doi: 10.3390/nu15102254, PMID: 37242136 PMC10223432

[27] Després J-P Lemieux I. Abdominal obesity and metabolic syndrome. Nature. (2006) 444:881–7. doi: 10.1038/nature05488, PMID: 17167477

[28] Sheng G Lu S Xie Q Peng N Kuang M Zou Y. The usefulness of obesity and lipid-related indices to predict the presence of non-alcoholic fatty liver disease. Lipids Health Dis. (2021) 20:134. doi: 10.1186/s12944-021-01561-2, PMID: 34629059 PMC8502416

[29] Song J-U Lee J Lim S-Y Gil H-I Chang Y Ryu S. Metabolically healthy and unhealthy obesity and the development of lung dysfunction. Sci Rep. (2023) 13:4938. doi: 10.1038/s41598-023-31960-7, PMID: 36973389 PMC10042802

[30] Zhou T Chen S Mao J Zhu P Yu X Lin R. Association between obstructive sleep apnea and visceral adiposity index and lipid accumulation product: NHANES 2015-2018. Lipids Health Dis. (2024) 23:100. doi: 10.1186/s12944-024-02081-5, PMID: 38600516 PMC11005189

[31] Shin K-A Kim Y-J. Usefulness of surrogate markers of body fat distribution for predicting metabolic syndrome in middle-aged and older Korean populations. DMSO. (2019) 12:2251–9. doi: 10.2147/DMSO.S217628 PMC6836308 31807040

[32] Bir A Ghosh A Chowdhury S. Visceral adiposity index and lipid accumulation product index: the promising role in assessing cardiometabolic risk in non-obese patients of PCOS. J Educ Health Promotion. (2023) 12:148. doi: 10.4103/jehp.jehp_1605_22, PMID: 37404927 PMC10317285

[33] Du T Yu X Zhang J Sun X. Lipid accumulation product and visceral adiposity index are effective markers for identifying the metabolically obese normal-weight phenotype. Acta Diabetol. (2015) 52:855–63. doi: 10.1007/s00592-015-0715-2, PMID: 25690647

[34] Liu Z Su Y Chen Q Xiao L Zhao X Wang F. Association of Dietary intake of vitamin E with chronic obstructive pulmonary disease events in US adults: a cross-sectional study of NHANES 2013-2018. Front Nutr. (2023) 10:1124648. doi: 10.3389/fnut.2023.1124648, PMID: 37125038 PMC10130507

[35] Xuan L Han F Gong L Lv Y Wan Z Liu H. Association between chronic obstructive pulmonary disease and serum lipid levels: a meta-analysis. Lipids Health Dis. (2018) 17:263. doi: 10.1186/s12944-018-0904-4, PMID: 30463568 PMC6249772

[36] Zhu Z Li J Si J Ma B Shi H Lv J. A large-scale genome-wide association analysis of lung function in the Chinese population identifies novel loci and highlights shared genetic aetiology with obesity. Eur Respir J. (2021) 58:2100199. doi: 10.1183/13993003.00199-2021, PMID: 33766948 PMC8513692

[37] Hegewald MJ. Impact of obesity on pulmonary function: current understanding and knowledge gaps. Curr Opin Pulm Med. (2021) 27:132–40. doi: 10.1097/MCP.0000000000000754, PMID: 33394747

[38] Park Y Kim J Kim YS Leem AY Jo J Chung K. Longitudinal association between adiposity changes and lung function deterioration. Respir Res. (2023) 24:44. doi: 10.1186/s12931-023-02322-8, PMID: 36750832 PMC9903501

[39] Goto Y Nagamine Y Hanafusa M Kawahara T Nawa N Tateishi U. Association of excess visceral fat and severe illness in hospitalized COVID-19 patients in Japan: a retrospective cohort study. Int J Obes. (2024) 48:674–82. doi: 10.1038/s41366-024-01464-z, PMID: 38233538

[40] Wang Z Zhou X Deng M Yin Y Li Y Zhang Q. Clinical impacts of sarcopenic obesity on chronic obstructive pulmonary disease: a cross-sectional study. BMC Pulm Med. (2023) 23:394. doi: 10.1186/s12890-023-02702-2, PMID: 37853348 PMC10585792

[41] Ravaut G Légiot A Bergeron K-F Mounier C. Monounsaturated fatty acids in obesity-related inflammation. Int J Mol Sci. (2020) 22:330. doi: 10.3390/ijms22010330, PMID: 33396940 PMC7795523

[42] Maurizi G Della Guardia L Maurizi A Poloni A. Adipocytes properties and crosstalk with immune system in obesity-related inflammation. J Cell Physiol. (2018) 233:88–97. doi: 10.1002/jcp.25855, PMID: 28181253

[43] Barnes PJ. Inflammatory mechanisms in patients with chronic obstructive pulmonary disease. J Allergy Clin Immunol. (2016) 138:16–27. doi: 10.1016/j.jaci.2016.05.011, PMID: 27373322

[44] Kotlyarov S Bulgakov A. Lipid metabolism disorders in the comorbid course of nonalcoholic fatty liver disease and chronic obstructive pulmonary disease. Cells. (2021) 10:2978. doi: 10.3390/cells10112978, PMID: 34831201 PMC8616072

[45] Charitos IA Aliani M Tondo P Venneri M Castellana G Scioscia G. Biomolecular actions by intestinal Endotoxemia in metabolic syndrome. Int J Mol Sci. (2024) 25:2841. doi: 10.3390/ijms25052841, PMID: 38474087 PMC10931779

[46] Skurikhin EG Pershina OV Pakhomova AV Pan ES Krupin VA Ermakova NN. Endothelial progenitor cells as Pathogenetic and diagnostic factors, and potential targets for GLP-1 in combination with metabolic syndrome and chronic obstructive pulmonary disease. Int J Mol Sci. (2019) 20:1105. doi: 10.3390/ijms20051105, PMID: 30836679 PMC6429267

[47] Jiang C Peng M Dai Z Chen Q. Screening of lipid metabolism-related genes as diagnostic indicators in chronic obstructive pulmonary disease. Int J Chron Obstruct Pulmon Dis. (2023) 18:2739–54. doi: 10.2147/COPD.S428984, PMID: 38046983 PMC10693249

[48] Rafie S Moitra S Brashier B. Association between the serum metabolic profile and lung function in chronic obstructive pulmonary disease. Turkish Thoracic J. (2018) 19:13–8. doi: 10.5152/TurkThoracJ.2017.17043, PMID: 29404181 PMC5783048

[49] Chan SMH Selemidis S Bozinovski S Vlahos R. Pathobiological mechanisms underlying metabolic syndrome (MetS) in chronic obstructive pulmonary disease (COPD): clinical significance and therapeutic strategies. Pharmacol Ther. (2019) 198:160–88. doi: 10.1016/j.pharmthera.2019.02.013, PMID: 30822464 PMC7112632

[50] Moreno-Vedia J Llop D Rodríguez-Calvo R Plana N Amigó N Rosales R. Lipidomics of triglyceride-rich lipoproteins derived from hyperlipidemic patients on inflammation. Eur J Clin Investig. (2023) 54:e14132. doi: 10.1111/eci.14132, PMID: 38010694

[51] Henein MY Vancheri S Longo G Vancheri F. The role of inflammation in cardiovascular disease. Int J Mol Sci. (2022) 23:12906. doi: 10.3390/ijms232112906, PMID: 36361701 PMC9658900

[52] Stafeev I Michurina S Agareva M Zubkova E Sklyanik I Shestakova E. Visceral mesenchymal stem cells from type 2 diabetes donors activate triglycerides synthesis in healthy adipocytes via metabolites exchange and cytokines secretion. Int J Obes. (2023) 47:732–42. doi: 10.1038/s41366-023-01317-1, PMID: 37100877

[53] Jubinville É Talbot M Bérubé J-C Hamel-Auger M Maranda-Robitaille M Beaulieu M-J. Interplay between cigarette smoking and pulmonary reverse lipid transport. Eur Respir J. (2017) 50:1700681. doi: 10.1183/13993003.00681-2017, PMID: 28889112

[54] Park K-H Shin D-G Cho K-H. Dysfunctional lipoproteins from young smokers exacerbate cellular senescence and atherogenesis with smaller particle size and severe oxidation and glycation. Toxicol Sci. (2014) 140:16–25. doi: 10.1093/toxsci/kfu076, PMID: 24798380

[55] Does AMVD Heijink M Persson LJ Kloos D Aanerud M Bakke P. Disturbed fatty acid metabolism in airway secretions of patients with chronic obstructive pulmonary disease. Eur Respir J. (2017) 50. doi: 10.1183/1393003.CONGRESS-2017.PA3913

[56] Agarwal AR Yin F Cadenas E. Short-term cigarette smoke exposure leads to metabolic alterations in lung alveolar cells. Am J Respir Cell Mol Biol. (2014) 51:284–93. doi: 10.1165/rcmb.2013-0523OC, PMID: 24625219

[57] Lykkesfeldt J. Malondialdehyde as biomarker of oxidative damage to lipids caused by smoking. Clinica chimica acta. (2007) 380:50–8. doi: 10.1016/J.CCA.2007.01.028, PMID: 17336279

[58] Wang L Iersel LEJ van Pelgrim CE Lu J Ark I van Leusink-Muis T., Effects of cigarette smoke on adipose and skeletal muscle tissue: in vivo and in vitro studies. Cells (2022) 11:2893. doi: 10.3390/cells11182893, PMID: 36139468 PMC9497292

[59] Knight-Lozano CA Young CG Burow DL Hu Z Uyeminami D Pinkerton K. Cigarette smoke exposure and hypercholesterolemia increase mitochondrial damage in cardiovascular tissues. Circulation. (2002) 105:849–54. doi: 10.1161/HC0702.103977, PMID: 11854126

[60] Wu K Luan G Xu Y Shen S Qian S Zhu Z. Cigarette smoke extract increases mitochondrial membrane permeability through activation of adenine nucleotide translocator (ANT) in lung epithelial cells. Biochem Biophys Res Commun. (2020) 525:733–9. doi: 10.1016/j.bbrc.2020.02.160, PMID: 32143825

[61] Crook S Büsching G Keusch S Wieser S Turk A Frey M. The association between daily exacerbation symptoms and physical activity in patients with chronic obstructive pulmonary disease. COPD. (2018) 13:2199–206. doi: 10.2147/COPD.S156986, PMID: 30140152 PMC6054763

[62] Salehidoost R Korbonits M. Glucose and lipid metabolism abnormalities in Cushing’s syndrome. J Neuroendocrinol. (2022) 34:e13143. doi: 10.1111/jne.13143, PMID: 35980242

[63] van Raalte DH Brands M van der Zijl NJ Muskiet MH Pouwels PJW Ackermans MT. Low-dose glucocorticoid treatment affects multiple aspects of intermediary metabolism in healthy humans: a randomised controlled trial. Diabetologia. (2011) 54:2103–12. doi: 10.1007/s00125-011-2174-9, PMID: 21562755 PMC3131514

[64] Higham A Lea S Simpson K Singh D. Lipids in the lung: respiratory inflammation in COPD. Eur Respir J. (2012) 40:385.

[65] Li X-F Wan C-Q Mao Y-M. Analysis of pathogenesis and drug treatment of chronic obstructive pulmonary disease complicated with cardiovascular disease. Front Med. (2022) 9:979959. doi: 10.3389/fmed.2022.979959, PMID: 36405582 PMC9672343

[66] Kotlyarov S Kotlyarova A. Anti-inflammatory function of fatty acids and involvement of their metabolites in the resolution of inflammation in chronic obstructive pulmonary disease. Int J Mol Sci. (2021) 22:12803. doi: 10.3390/ijms222312803, PMID: 34884621 PMC8657960

